# Intra-operative measurement of tumour size in breast cancer and its comparison with other methods: a prospective study

**DOI:** 10.3332/ecancer.2008.96

**Published:** 2008-09-26

**Authors:** VP Verma, N Kaur, N Agarwal, SK Bhargava, UR Singh, S Saha, A Raheja

**Affiliations:** 1 Department of Surgery, University College of Medical Sciences and Guru Teg Bahadur Hospital, Delhi-110095, India; 2 Department of Radiodiagnosis, University College of Medical Sciences and Guru Teg Bahadur Hospital, Delhi-110095, India; 3 Department of Pathology, University College of Medical Sciences and Guru Teg Bahadur Hospital, Delhi-110095, India

## Abstract

Accurate measurement of breast tumour size determines staging and prognosis. Discrepancies amongst clinical examination (CE), ultrasonography (USG), mammography, pathological examination (PE) and magnetic resonance imaging have been reported. However, few studies have evaluated changes in breast tumour size from the operating table to the laboratory.

**Objectives and methods:**

A prospective study was designed to assess the intra-operative (IO) tumour size in 29 patients of breast cancer presenting to a tertiary care centre in Delhi and to compare it with CE, USG and PE.

**Observations and results:**

Twenty-nine patients (mean age: 47 years), presenting with invasive duct carcinoma (stage IIIA: 31%, stage IIB: 28%), were included in the study. Comparison with mean IO (4.2 cm) revealed that both USG and PE underestimated tumour size by a mean of 0.35 cm (8.4%) and 0.45 cm (10.7%), respectively, in most patients. CE tended to overestimate size by 0.82 cm (19.8%). All three modalities showed statistically significant correlation with IO (maximum Pearson’s correlation coefficient for PE=0.937, p<0.001; *R*^2^=0.877, maximum for PE). Two-way analysis of variance revealed mean difference in size to be statistically significant (p=0.000) only between CE and IO.

**Discussion:**

Formalin processing causes changes in tumour dimensions in the breast, causing reduction in tumour size. It may also have a bearing on the assessment of surgical margins in breast conservation surgery. Immediate post-operative measurement of the specimen is ideal. Protocols for specimen fixation should be standardized.

## Background

Tumour size is an independent prognostic factor in breast carcinoma and is a good predictor of lymph node metastases. Change in tumour size, as a response to chemotherapy, is also an independent predictor of disease-free survival [1,2]. The measurement of tumour size should be accurate as even small discrepancies can affect staging and treatment. The various techniques to assess tumour size are clinical examination (CE), mammography (MG), ultrasonography (USG), magnetic resonance imaging and pathologic examination (PE). The last is still considered the blueprint for pathological/final staging and for formulation of the treatment plan.

It is known that there is considerable discrepancy in size after formalin processing in many cancers. Uterine, arterial, colorectal, lung and prostate have all been shown to undergo shrinkage in one or more dimensions, and even volume [3–7]. In breast cancer, variation has been reported between imaging modalities (USG, MG), PE and CE [8–10]. There are no guidelines regarding the state of the specimen to be taken as the standard for staging, that is fresh or fixed. The three-dimensional measurements are most easily and accurately obtained from the fresh gross specimen. [7,11], but constraints of time and resources compel most laboratories to obtain the specimen after formalin fixation. Several reasons have been attributed to the disharmony in size between fresh and fixed specimens, including the use of formalin, the ‘pancake phenomenon’, compression during specimen radiography, tissue composition of the tumour, histological subtype [12–14].

The number of studies which have attempted to quantify the extent of this size discrepancy is surprisingly low [8,9,12,14] and even fewer have suggested corrections [9].

It is evident that studies addressing change in size should be organ-specific and account for tissue constitution. In this study, we intra-operatively (fresh state) measured the tumour size in patients with breast carcinoma and, considering it as the actual size, compared it to CE, USG and PE. We also attempted to predict the actual size from other measurements, using statistical formulae.

## Materials and methods

A prospective study was carried out at the departments of surgery and pathology at the University College of Medical Sciences and Guru Teg Bahadur Hospital, Delhi, India from December 2006 to March 2008. Twenty-nine patients with a palpable breast lump diagnosed to be cancer, and planned for surgery, were enrolled for the study. Patients without a definite palpable breast lump, and those with inflammatory carcinoma, were excluded.

A detailed history was obtained regarding patients’ complaints and history of known risk factors such as family history of breast or ovarian cancer, previous breast disease, radiation exposure, reproductive details and drug therapy.

Physical examination, chest x-ray, and USG of the breast for tumour size were carried out on all patients. The latter was done by an experienced radiologist to minimize operator variation. For patients with locally advanced disease, imaging for metastases in the form of a bone scan and USG of the abdomen was also performed.

The accuracy in measurement was given maximum importance, and all the patients were seen by the same team (surgeon and radiologist) to avoid inter-observer variation.

## Clinical assessment of breast tumour size

Clinically, the breast lump diameter was measured in two dimensions with Vernier’s calipers, and tumour size was taken as the maximum diameter.

## Ultrasonographic assessment of tumour size

A high-resolution linear array ultrasonic transducer with a frequency 7.5 MHz was used to assess the breasts mainly for tumour size, and for associated findings such as satellite nodules. Here, the measurements were taken in three dimensions, and the maximum diameter was recorded as tumour size.

The assessment of the patient was completed as close to the proposed operation as feasible (2–3 days maximum), to avoid changes in the observations.

## Intra-operative assessment of tumour size

After completing the proposed operation (modified radical mastectomy), the specimen was examined by the same surgeon who had performed the clinical examination. The specimen was sliced with the help of a scalpel, by cutting longitudinally from the posterior aspect into slices approximately 2 cm thick. Maximum tumour diameter in three dimensions was measured to the nearest millimeter, and labelled as tumour size. To avoid problems in reporting due to the slicing affecting the deep-resected margin, tissue from deep within the tumour specimen was excised and labelled separately for histopathological examination.

## Pathological assessment

The surgical specimen was fixed in formalin (for 14–20 hours) after intra-operative recording of tumour size, and sent for histological examination. The pathologist also recorded the size in three dimensions.

Follow-up advice to patients was in accordance with the accepted protocol for breast carcinoma.

## Statistical analysis

The maximum diameters measured by the different methods were compared, and the difference between individual data and the mean difference was calculated. Using two-way analysis of variance, taking size as a dependent variable, and the measurement method and subject as independent factors, post-hoc pairwise comparison was done using Bonferroni adjustment for calculating significant value of the mean and the difference of means.

Pearson’s method was used to calculate correlation between maximum sizes measured by all four methods. Plotting a graph, and identifying the line of best fit by linear regression obtained correlation between intra-operative, clinical, ultrasonographic and pathological size. Significance value *R*^2^ was derived from differences between the line of best fit and the line of equality.

Using stepwise multiple regressions, taking the intra-operative measurement as dependent, and clinical, ultrasonographic and pathological measurements as independent predictors, an estimation of the intra-operative measurement was achieved using the other three methods.

## Observations and results

A total of 29 patients were included in the study. The patients had a mean age of 47 years (range of 31–70 years). Most patients were 31–50 years of age. All patients had a lump in one breast. Three patients complained of associated pain and two of bloody nipple discharge. Only one patient had a history of associated risk factors, that is mastectomy for contralateral breast cancer two decades previously. Seventeen (58.6%) patients had a tumour located in the upper outer quadrant of the breast, while two had a large tumour occupying all four quadrants.

The stage-wise distribution (clinical and pathological) of patients is shown in Table 1. None of the patients presented in clinical stage I. On pathological staging, most patients were in stage IIIA (31.02%) or stage IIB (27.6%). Twenty-four (82.76%) patients had invasive breast cancer of intermediate histological grade, that is Grade 2.

## Comparison between measurements of tumour size according to the different methods

### (1) Clinical versus intra-operative measurement of tumour size

The mean diameter on clinical measurement was 4.98 cm and on intra-operative measurement was 4.16 cm. Hence, clinical examination tended to overestimate the tumour size by 19.76% (mean 0.82 cm). In 24 patients (82.75%), tumour size was overestimated by clinical examination, and in only five patients it was of the same value as intra-operative.

### (2) Ultrasonography versus intra-operative

The mean maximum diameter (tumour size) by USG was 3.81 cm, while intra-operative measurement was 4.16 cm. The mean difference was 0.35 cm, and an underestimation by USG of 8.38%. Only in five patients did USG overestimate the size of the tumour.

### (3) Pathological versus intra-operative

Pathological measurement tended to underestimate the tumour size. In seven specimens (24.14%), pathological measurement was the same value as intra-operative, but in 22 (75.86%), it underestimated the size (range 0–2.5 cm). The mean pathological size was 3.71 cm, as compared to 4.16 cm intra-operatively (mean difference 0.45 cm, 10.7%).

Using Pearson’s correlation co-efficient, pathological measurement was found to have maximum correlation with intra-operative measurement (co-efficient 0.937; p<0.001). Clinical measurement and USG also showed good correlation (0.790; p<0.001 and 0.877; p<0.001, respectively.)

**Figure 1:**
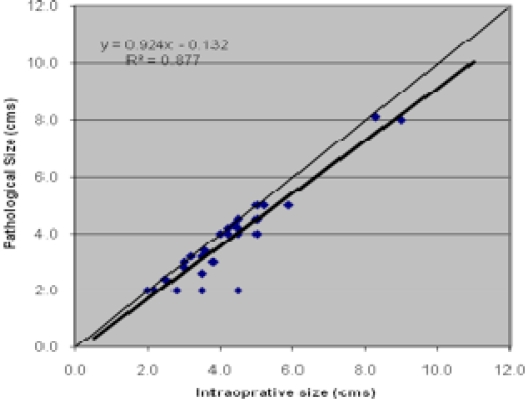
Correlation between intra-operative and pathological size

Comparison between intra-operative and the other methods was also done by plotting a graph, and obtaining line of best fit by linear regression. Significance value *R*^2^ was derived from differences between the line of best fit and the line of equality. This also showed that pathological measurement had maximum correlation with intra-operative size (Figure 1).

Using two-way analysis of variance, taking size as the dependent variable and measurement method and subjects as independent factors, post-hoc pairwise comparison was done using Bonferroni adjustment (for multiple comparisons). It was seen that the mean difference in size was statistically significant (p=0.000) only for comparison between intra-operative and clinical measurement.

## Prediction of intra-operative measurement by other methods

Using stepwise multiple regression taking intra-operative measurement as dependent, and clinical, USG and pathological measurement as independent predictors, the following equations were derived:

(1) Intra-operative size = 0.324 cm + (0.611 × pathological size) + (0.301 × clinical size)

(2) Intra-operative size = 0.447 cm + (0.731 × clinical size)

Hence, by using the clinical and pathological tumour size, we can predict the intra-operative size.

## Discussion

The demographic profile of our patients is similar to other Indian data [15–17]. Most of our patients present with locally advanced breast cancer, mainly due to socio-cultural reasons. For the same reasons, they also prefer to opt for mastectomy, as frequent follow-up from far-flung areas can be prohibitive. This could explain the performance of modified radical mastectomy in all the 29 patients.

All the modalities used in our study have proven to be as accurate as suggested in the literature. Clinical examination was compared with pathological examination as early in 1983, and some ‘adjustment’ was attempted for skin and fat thickness, as it tends to result in overestimating the size [18]. CE has been found to be very accurate for larger tumours, and many centres do not perform routine imaging for size (USG) in large breast tumours. A good correlation was seen between intra-operative tumour size and the USG measurement in our patients. USG is an excellent investigative tool for dense breasts, deep-seated lesions, and to follow the response to chemotherapy. USG is also considered superior to mammography for pre-operative estimation of tumour size [10,19]. This is also our experience; USG is easy to use and expertise is available at our hospital. Hence, we did not include mammography as a measurement method in the present study. Despite these advantages, USG tends to underestimate the tumour size when compared with pathologic size (the ‘gold standard’) in most cases [8,9,19–21]. Bosch and colleagues found that USG is an accurate predictor of pathological size, and pathological size can be determined by size measured by ultrasound + 3 mm [19]. In our study, we found a mean difference of 3.48 mm, which is comparable, but our gold standard for comparison was considered to be the intra-operative size. Recently, a lot of interest has been created by the use of the contrast-enhanced USG. In comparison to conventional USG, it has been shown to be a more accurate measure for pre-operative tumour size in invasive ductal cancer of the breast [22,23].

Despite the importance of tumour size in staging, management, choice of operation and prognosis, the exact state of the specimen to be measured has not been standardized. Formalin processing has been shown to alter tumour dimensions in many solid organ cancers, including the breast [3–7,12–14]. The effect of this alteration, chiefly reduction in size, has been shown to have worrisome implications for analysis of the margins, especially in breast conserving surgery [12,14]. Yeap *et al* have shown that loss of margins due to fixation can be substantial enough to influence re-excision decisions. They have thus recommended suspension of the specimen during transport, as well as stressing the need for more guidelines for specimen handling, to minimize these errors [14,24]. From our data, treatment decisions were unlikely to be influenced by small errors in margins, as all were mastectomy specimens. Also, there were no reported ‘close’ margins however, as a larger percentage of our patients are expected to undergo breast-conserving therapy in the near future, these issues will be very relevant. Many authors have identified factors, which affect the variation in size after fixation, such as histology, manipulation of specimen and the size of the tumour itself. Due to similar stage and histology of almost all our patients, this variation could be expected to be minimal. A single study by Pritt *et al* also states that overnight fixation does not change the gross tumour size, as long as microscopic measurements are not considered for comparison [13]. There is thus controversy over selection of the ‘actual’ size for staging. Most previous studies have taken pathologic size as the gold standard.

Formalin fixation of tissue specimens occurs in two phases. During the first phase, the fixative penetrates the tissue by diffusion and accumulates to a sufficient concentration. In the second phase, formalin exhibits a gelling action and chemically binds to protein amino groups, eventually leading to extensive cross-links between proteins or between proteins and nucleic acids [25,26]. Although formalin is used to stabilize and preserve tissue ultrastructure, it occasionally causes distortion, vacuolization and cell shrinkage. These reactions not only affect tissue histochemical reactivity but also possibly cause gross change in specimen shape and size. Delayed fixation (>30 minutes) increases proteolytic degradation and may affect tumour-size measurement. The fixation duration also has an effect. Short fixation times have an effect only at the periphery of the tissue block, whereas prolonged fixation (>24 to 48 hours) leads to excessive structural changes. Many studies have documented this from time to time. In our patients, the duration of the specimen in formalin ranged from 14–20 hours.

In two other similar studies carried out in breast carcinoma [9,14], all the measurements were recorded by the pathologist. This may be technically sound, but it may bring in some observer bias. In our hospital, the laboratory receives a large number of specimens, and the pathologist is unable to measure each specimen prior to fixation. For these reasons, we recruited three constant teams (surgeon, radiologist and pathologist) to minimize the observer variability. Both the previous studies have reported a discrepancy of only 1–2 mm in intra-operative and pathological tumour size, but even so in only a few patients. On the other hand, in our study, we found a mean difference of 0.44 cm in 75.86% of patients. The reasons behind this may be the greater delay in fixation, delay in measurement after fixation (prolonged fixation) or measurement by different persons (the surgeon and the pathologist). This study may thus serve as a pilot study to a larger study, which could include all types of tumour excisions and follow standard guidelines for specimen processing.

Estimation of the accurate tumour size by deriving formulae from other modalities of tumour size estimation (e.g. estimation of pathological tumour size from that measured from USG) has been attempted by a few workers previously [19]. The equations that we have derived may also be useful, provided that they are validated by a larger amount of data.

## Conclusions

The tumour-size measurement is affected by factors such as specimen handling, delay in tumour fixation, duration of formalin fixation. Keeping in mind the great importance of tumour size in the management of breast cancer, the protocols used to measure size should be standardized to minimize chances of error in measurements and to bring uniformity in data analysis. Current data suggests that immediate fixation and 6–8 hours of formalin immersion does not affect the tumour size measurement. Larger studies are required to validate this conclusion.

## Figures and Tables

**Table 1:**
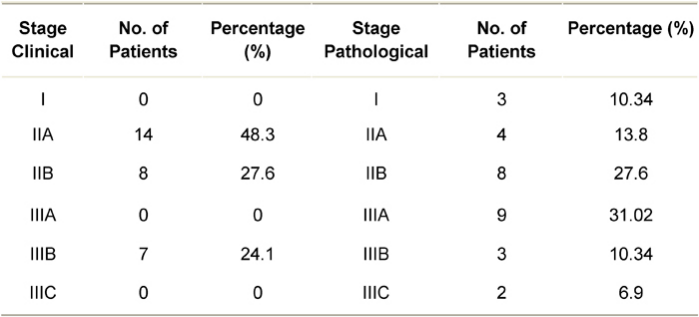
Distribution of breast cancer patients by clinical and pathological stage
